# Management of Massive Post‐Tracheostomy Subcutaneous Emphysema With an Adjustable‐Length, Wire‐Reinforced Tracheostomy Tube: A Case Report

**DOI:** 10.1002/ccr3.73490

**Published:** 2026-09-06

**Authors:** Omar Aboul Hosn, Rita Khattar, Jaafar A. Hadi, Gabriel Dunya, Sami Jean Melki

**Affiliations:** ^1^ Department of Otolaryngology‐Head and Neck Surgery American University of Beirut Medical Center Beirut Lebanon; ^2^ Department of Diagnostic Radiology American University of Beirut Medical Center Beirut Lebanon

**Keywords:** adjustable‐length tracheostomy tube, Pneumomediastinum, subcutaneous emphysema, Tracheomegaly, Tracheostomy

## Abstract

Massive subcutaneous emphysema with pneumomediastinum is a rare but potentially life‐threatening complication following tracheostomy, for which standardized management strategies are lacking. We report the case of a 67‐year‐old man who developed progressive cervicofacial and thoracic subcutaneous emphysema on postoperative Day 9 after an otherwise uncomplicated surgical tracheostomy using a fenestrated tube. The tracheostomy had been performed after multiple failed extubation attempts due to Type 2 respiratory failure in the setting of interstitial lung disease. Diagnostic evaluation with fiberoptic tracheoscopy and computed tomography of the neck and chest demonstrated extensive subcutaneous emphysema, pneumomediastinum, tracheal dilation, a suspected persistent air leak related to tube–trachea size mismatch, and the presence of fenestrations. Initial conservative bedside measures failed to halt progression. Subsequent exchange to a non‐fenestrated, flexible, wire‐reinforced cuffed tracheostomy tube with adjustable length resulted in rapid regression and complete resolution of the emphysema without the need for further intervention. This case highlights the importance of early recognition of anatomical and device‐related contributors to post‐tracheostomy air leak and demonstrates that prompt, targeted tracheostomy tube selection can provide effective airway sealing and obviate invasive management strategies.

## Key Clinical Message

Massive post‐tracheostomy subcutaneous emphysema with pneumomediastinum may result from persistent peri‐tubal air leak, particularly in patients with tracheomegaly. In this setting, early exchange to a non‐fenestrated adjustable‐length tracheostomy tube can optimize airway sealing, improve intratracheal positioning, control ongoing air leakage, and potentially avoid invasive decompressive interventions.

## 1 Introduction

Tracheostomy is a surgical airway procedure that involves creating an opening in the anterior tracheal wall to secure ventilation and airway access. It is commonly performed in patients with acute respiratory failure following prolonged endotracheal intubation, upper airway obstruction, anticipated difficult airway, or inability to manage copious secretions [1]. Early perioperative complications include hemorrhage, pneumothorax or pneumomediastinum secondary to false tract formation, subcutaneous emphysema, esophageal injury, and tracheal ring fracture. Late complications are typically associated with long‐term tracheostomy use and include airway stenosis, granulation tissue formation, tracheomalacia, and the development of tracheoesophageal or tracheoinnominate fistulas [2].

Subcutaneous emphysema (SE) is a recognized complication of tracheostomy, with reported incidence rates of up to 9% [3]. SE refers to the abnormal accumulation of air within the subcutaneous tissues. Its pathophysiology follows one of two mechanisms: endogenous gas production, as seen in necrotizing infections, or air dissection into tissue planes from internal or external sources of pressurized air [4]. The latter mechanism is more frequently encountered and is associated with conditions such as pneumothorax, pneumomediastinum, and laryngotracheal injury.

Based on a literature review of PubMed, Scopus, and EMBASE using the keywords “subcutaneous emphysema,” “pneumomediastinum,” “fenestrated tracheostomy tube,” “tracheomegaly,” and “peri‐tubal air leak”. Only a limited number of reports describe massive subcutaneous emphysema with concomitant pneumomediastinum following tracheostomy. However, a standardized management approach has not been established. In this report, we describe a patient who developed massive SE of the head and neck with pneumomediastinum several days after insertion of a conventional fenestrated tracheostomy tube.

To our knowledge, this is one of the few reported cases in which massive post‐tracheostomy subcutaneous emphysema associated with tracheomegaly, a fenestrated tracheostomy tube, and a stomal subcutaneous breach was successfully managed primarily through targeted tracheostomy tube redesign using an adjustable‐length, wire‐reinforced system, without the need for invasive decompressive measures.

Written informed consent was obtained from the patient for publication of this case report and any accompanying images.

## 2 Case Presentation

A 67‐year‐old man with idiopathic pulmonary fibrosis (IPF), hypertension, a history of nasal basal cell carcinoma excision, depression on medical therapy, significant coronary artery calcifications, and a recent admission for medically treated pneumonia re‐presented with hypoxemia and increased work of breathing.

He was diagnosed with hospital‐acquired pneumonia due to methicillin‐sensitive *Staphylococcus aureus* , complicated by acute Type 2 respiratory failure requiring intensive care unit admission and bilevel positive airway pressure (BiPAP). After multiple failed weaning attempts, even for minimal periods of time, our team was consulted for tracheostomy placement.

The patient underwent an uncomplicated surgical tracheostomy performed by an attending physician with the assistance of a fourth year resident. Limited dissection and soft tissue manipulation was performed in all planes. A Shiley size 8 fenestrated tracheostomy tube (Shiley tracheostomy tube (Covidien, Mansfield, MA, USA)) was placed between the first and second tracheal rings using a Björk flap technique, consistent with routine institutional practice. The cuff was subsequently inflated with 10 mL of air. A single stay suture using 4–0 Vicryl was placed, and the tracheostomy tube was secured to the skin using four 2–0 silk sutures and a tracheostomy tie.

The decision to use a fenestrated tracheostomy tube was guided by the family's desire for phonation after extubation, as well as financial considerations, given that insurance does not cover frequent tracheostomy tube changes; consequently, tube exchanges are performed on a monthly basis. The postoperative course was initially unremarkable. A routine postoperative chest radiograph showed an adequately positioned tube 5 cm above the carina. Skin sutures were subsequently removed on postoperative Day 7 (POD‐7). A 2 × 2‐in. gauze dressing was placed under the superior and inferior flanges of the tracheostomy tube. These two interventions are part of our routine postoperative care. On POD‐9, our team was re‐consulted for progressive SE. The patient was awake, hemodynamically stable, and without respiratory distress. The patient was on assist‐control ventilation. Ventilator requirements were minimal (Tidal volume 450 mL, positive end‐expiratory pressure [PEEP] 5 cm H_2_O, fraction of inspired oxygen [FiO_2_] 40%). However, the exhaled tidal volumes remained consistently low, typically ranging between 150 and 180 mL, strongly suggesting the presence of a significant air leak. Examination revealed extensive SE with palpable crepitus involving the neck bilaterally and extending to the cheeks and lower eyelids. The tracheostomy stoma was well healed; however, the tube was excessively mobile, with the fenestra abutting the superior edge of the stoma.

**FIGURE 1:**
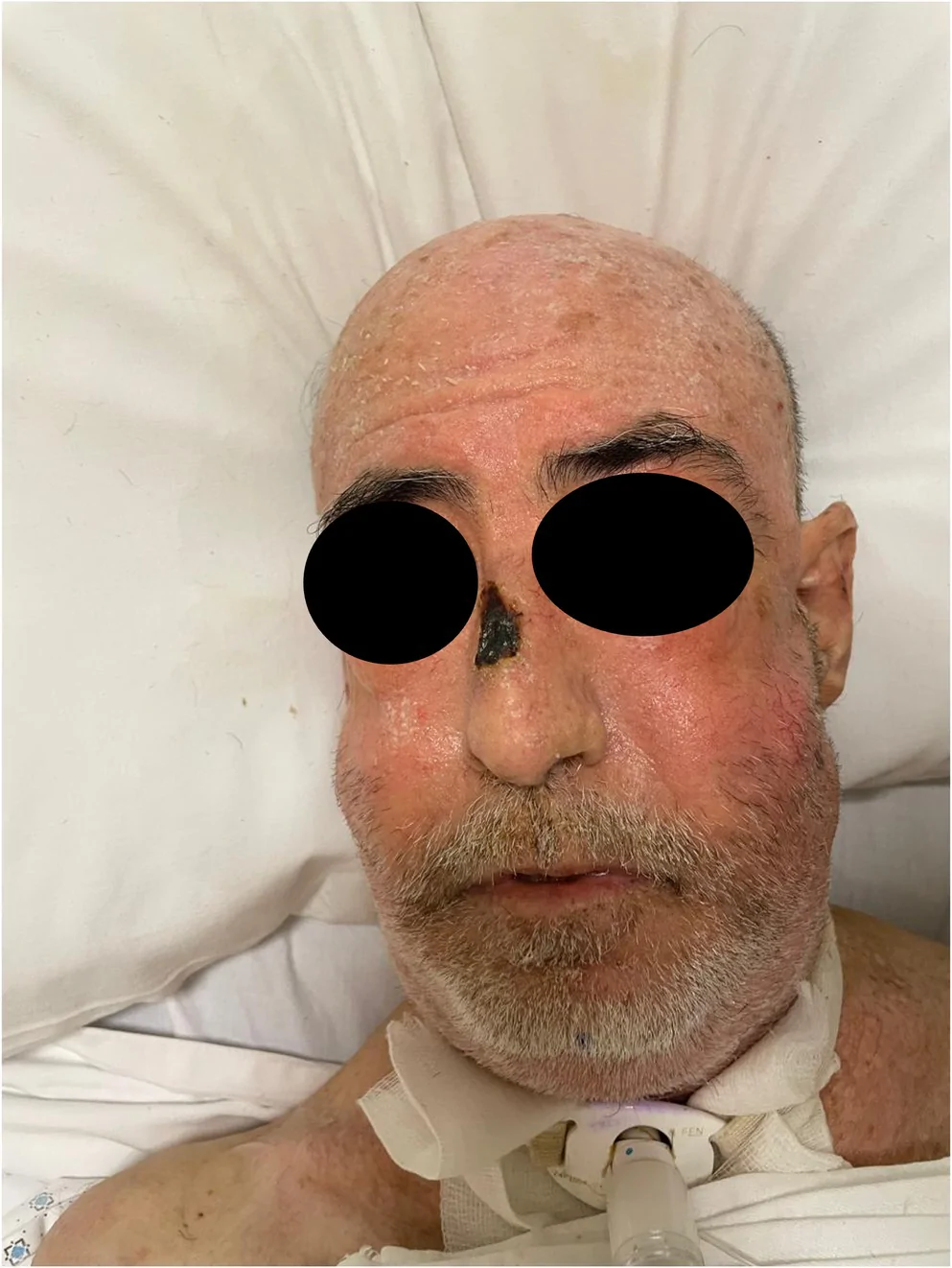
Clinical photograph of the patient's face demonstrating subcutaneous emphysema, with visible swelling extending to the periorbital region and involving the upper eyelids.

Flexible fiberoptic tracheoscopy through the tracheostomy tube demonstrated a patent airway with clear visualization of the carina and no evidence of intraluminal obstruction, tracheomalacia, or tracheal injury. Chest radiography revealed no pneumothorax. Initial management included optimization of tracheostomy positioning, cuff pressure and function assessment, and close observation. It should be noted that cuff pressure checks are routinely performed twice daily by specialized respiratory therapists, ensuring adequate pressure (between 20 and 25 cm H_2_O) and proper function. Despite these measures, SE progressed over the following 2 days, extending to the parietal scalp while the patient remained clinically stable (Figure 1).

**FIGURE 2:**
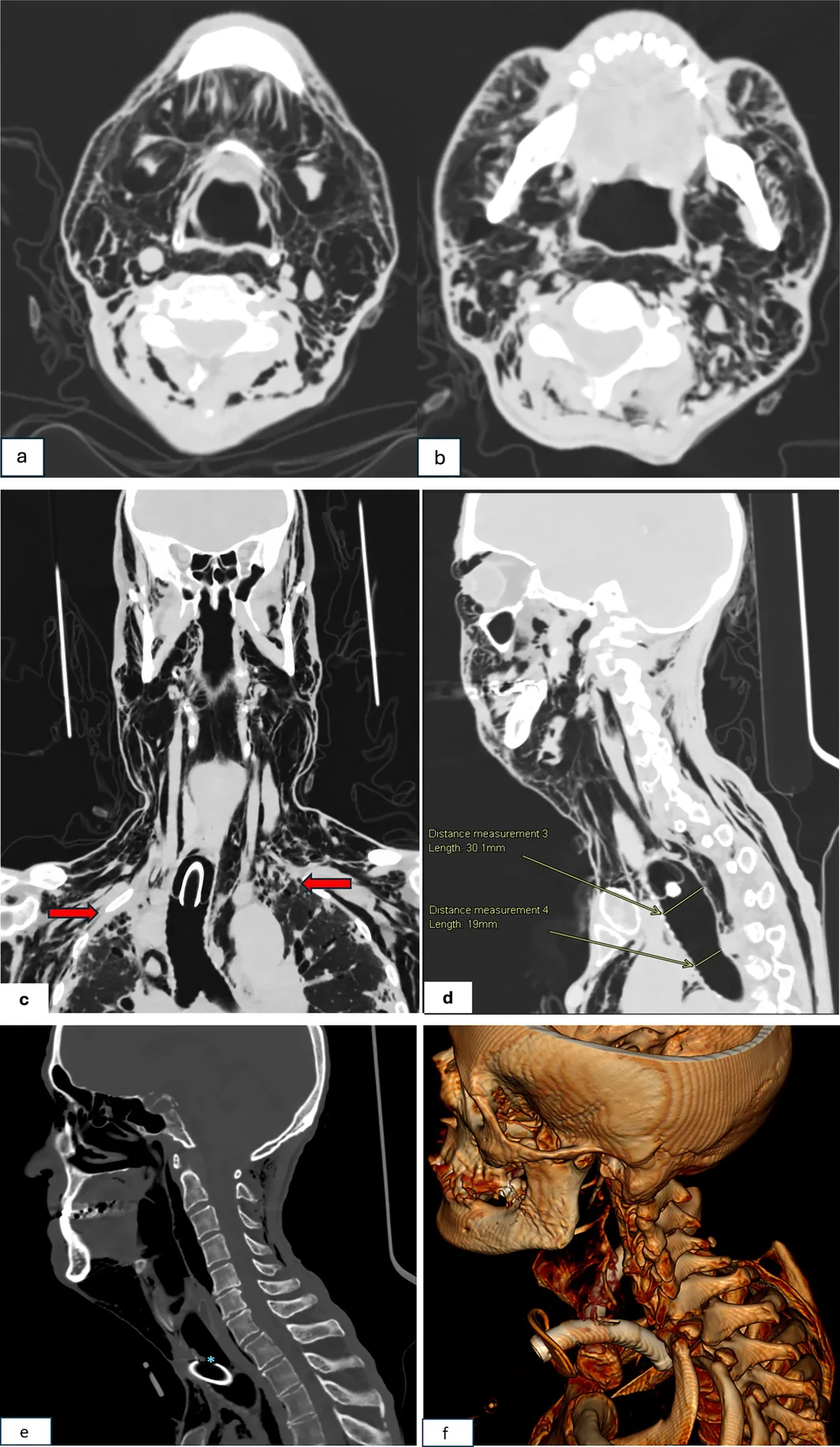
(a, b) Axial CT images of the neck at the oropharyngeal (a) and nasopharyngeal (b) levels demonstrate extensive subcutaneous emphysema with diffuse air dissecting throughout the superficial and deep neck spaces bilaterally. (c, d) Coronal CT of the neck and chest (c) demonstrates extensive cervicothoracic subcutaneous emphysema without evidence of pneumothorax at the lung apices (red arrows). Sagittal CT (d) demonstrating proximal tracheal widening with gradual reduction in caliber inferiorly. On this cut, the estimated anteroposterior tracheal diameter measures approximately 30 mm superiorly compared to approximately 19 mm inferiorly. (e, f) Sagittal CT images of the neck, bone window (e) and 3D reconstruction (f), show the upper fenestration of the tracheostomy positioned at the level of the anterior tracheal wall (blue asterix), with air extending into the surrounding soft tissues, suspicious for peri‐tubal air leakage. No focal tracheal wall defect.

Given the progression of SE, computed tomography (CT) of the neck and chest was obtained, demonstrating diffuse SE involving the chest wall, mediastinum, superficial and deep cervical, retropharyngeal and prevertebral spaces, with extension into the orbits and temporal regions. The tracheostomy tube tip was positioned 7.5 cm above the carina, with an enlarged tracheal diameter measuring 26–40 mm, likely related to tractional airway remodeling in the setting of advanced fibrotic lung disease. The fenestra was predominantly intraluminal, with partial abutment against the anterior tracheal wall and adjacent soft tissues. No discrete tracheal defect was identified. (Figure 2a–f).

The thoracic surgery team was consulted and recommended no intervention from their standpoint, as imaging did not demonstrate pneumothorax and the patient's clinical presentation was considered to be primarily related to the tracheostomy tube.

**FIGURE 3:**
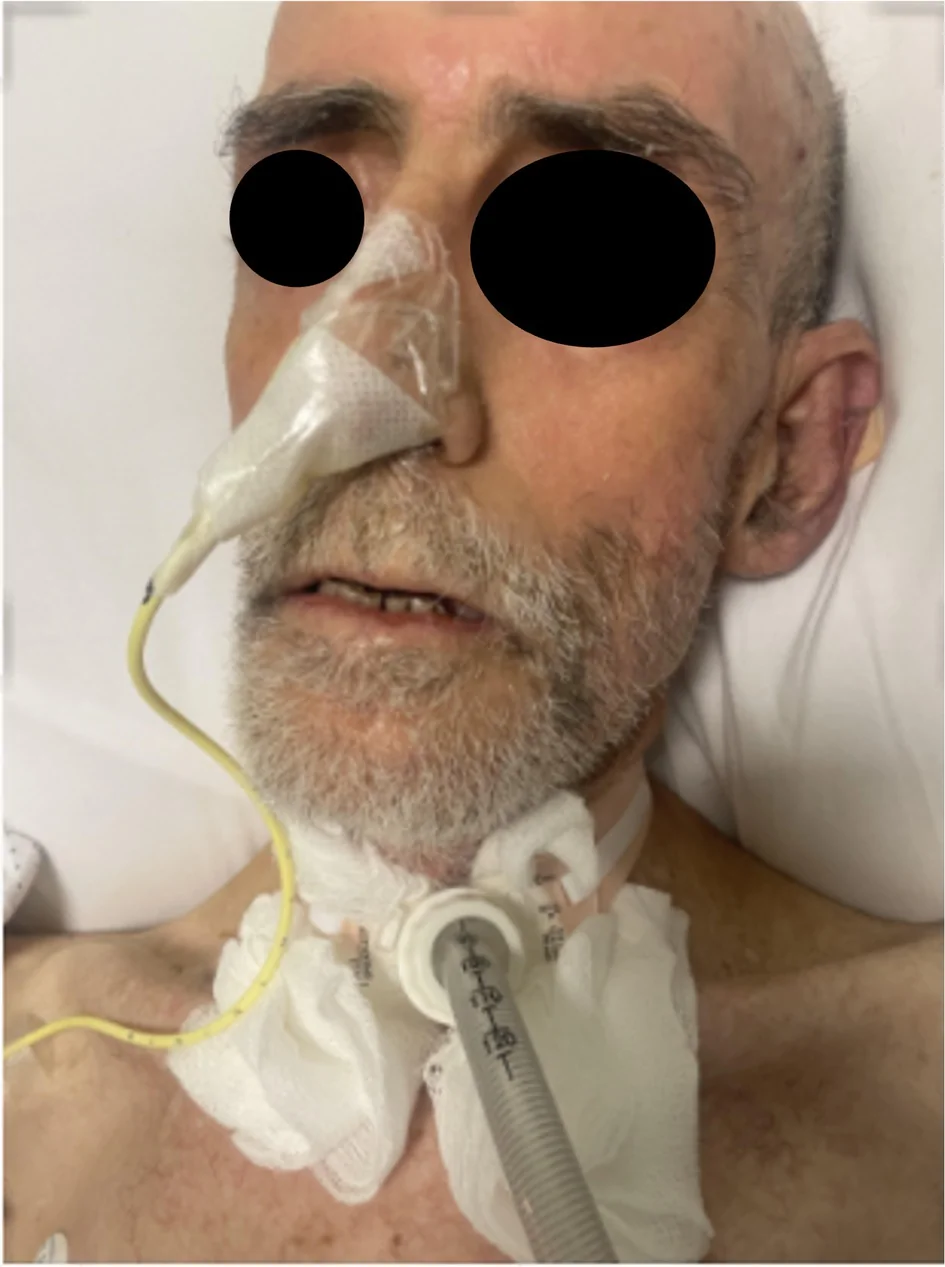
Post–tracheostomy tube exchange demonstrating marked resolution of subcutaneous emphysema involving the face and periorbital regions. The newly placed adjustable‐length tracheostomy tube is clearly visible and appropriately positioned.

Based on these findings, supplemental oxygen was increased to approximately 60% FiO_2_ overnight to promote nitrogen washout and facilitate resorption of subcutaneous air; however, no clinical improvement was observed. In the setting of extensive SE, a relatively large tracheal diameter, the presence of fenestrations, and failure of supportive measures, tracheostomy tube exchange was planned. On POD‐15, the patient was taken to the operating room, where inspection of the stoma revealed a localized breach in the subcutaneous tissues at the superior aspect of the wound flange, without evidence of posterior tracheal wall injury. The fenestrated tube was replaced with a Spiraflex size 9, non‐fenestrated, adjustable‐length, wire‐reinforced cuffed tracheostomy tube (Spiraflex tracheostomy tube (Smiths Medical, Minneapolis, MN, USA)) with the same outer diameter (Table 1). The tube length was adjusted to 95 mm. The cuff was inflated to 25 cm H_2_O, measured with a manometer, and the tracheostomy ties were readjusted to improve stabilization. Postoperatively the SE began to regress within 3 days and resolved completely without recurrence (Figure 3). The cuff pressure remained stable and ventilator parameters improved. Air leakage remained undetectable. The final tube position remained unchanged as confirmed by daily postoperative chest radiography. Unfortunately, 1 month after the procedure the patient passed away due to superimposed ventilator‐associated pneumonia progressing to septic shock.

**TABLE 1:** Published case reports of post‐tracheostomy subcutaneous emphysema.

Author (Year)	Age	Sex	Key findings	Intervention
Mostert (2001)	Not specified	Not specified	Subcutaneous emphysema caused by air leakage through a fenestrated tracheostomy tube	Fenestrated tube removed and replaced with a non‐fenestrated tube
Showmaker (2010)	47	Female	Life‐threatening tension subcutaneous emphysema after open tracheostomy, no pneumothorax	Multiple 2‐cm chest and abdominal skin incisions with manual evacuation of subcutaneous air
Elkholy (2019)	84	Female	Massive subcutaneous emphysema with pneumomediastinum and pneumoperitoneum after tracheostomy tube repositioning	High‐FiO_2_ mechanical ventilation, ICU monitoring; no surgical repair performed
Alshoubi (2022)	64	Female	Extensive cervicofacial subcutaneous emphysema after open tracheostomy due to tight tracheostomy ties	Tracheostomy ties loosened; IV sedation initiated
Kim (2023)	61	Female	Subcutaneous emphysema and pneumomediastinum due to stomal dehiscence after emergent tracheostomy	Surgical closure of stomal defect + negative‐pressure wound therapy (100 mmHg)

Abbreviations: cm, centimeters, FiO_2_, fraction of inspired oxygen, ICU: intensive care, IV: intravenous, mmhg, millimeter of mercury.

## 3 Discussion

SE is a relatively rare complication of tracheostomy, typically occurring within hours to days following the procedure. Predisposing factors include extensive tissue dissection, tracheostomy tube obstruction, high‐pressure mechanical ventilation, tracheal wall injury [5], the use of fenestrated tracheostomy tubes [6], and percutaneous tracheostomy techniques [7]. Following tracheostomy, SE most commonly results from air leakage due to cannula malposition or displacement. In most cases, SE is managed conservatively, as it is generally benign and self‐limiting, with spontaneous air resorption. However, when SE is extensive or associated with pneumomediastinum, it may progress to a life‐threatening condition with acute cardiovascular compromise, necessitating prompt and more aggressive intervention [8].

The primary goal in managing SE is effective decompression of the thoracic inlet and cervical soft tissues through identification and correction of the source of air leakage, leading to gradual resolution of the emphysema [3]. Several invasive strategies have been described for severe or refractory cases, including multiple subcutaneous “gill slit” incisions to relieve tension SE, as reported by Showmaker et al. [9], and negative‐pressure wound therapy combined with surgical closure of tracheostomal dehiscence, as described by Kim et al. [10].

In contrast, conservative strategies have also been described. El Shoubi et al. reported clinical improvement following simple release of tracheostomy tie tension, thereby relieving external compression and facilitating air egress [11]. El Kholy et al. managed their case with administration of 100% inspired oxygen for 48 h, promoting nitrogen washout, increasing the diffusion gradient for gas resorption, and accelerating air absorption by approximately 4‐ to 6‐fold [12].

In our case, a possible interplay between anatomical and device‐related factors likely contributed to the development of massive SE. Normal adult tracheal diameters measure < 25 mm in men and < 21 mm in women. Tracheobronchomegaly, or Mounier–Kuhn syndrome, is diagnosed when the tracheal diameter exceeds 30 mm on CT imaging [13]. In our case, the markedly enlarged tracheal diameter observed on imaging, likely related to the patient's IPF, resulted in a size mismatch with the initially placed regular‐length Shiley size 8 tracheostomy tube, allowing air to dissipate from around the tube [14]. After removal of the skin sutures, the mobility of the tracheostomy tube increased, resulting in friction against the superior wound margin. Such movement may have caused a breach in the subcutaneous tissues, facilitating air leakage, particularly given its direct correspondence with the site of the tube fenestration. In addition, the presence of fenestrations, despite the use of a non‐fenestrated inner cannula, likely facilitated air leakage [15, 16]. Escaping air likely passed through a partial dehiscence at the superior aspect of the stoma and dissected superiorly along the cervical soft tissue planes, leading to progressive SE. In the most closely related reported case by Mostert et al., SE developed acutely within minutes following percutaneous tracheostomy and was managed by replacing a fenestrated tracheostomy tube with a non‐fenestrated tube of the same size [6] Table 2.

**TABLE 2:** Comparison of conventional and adjustable‐length tracheostomy tubes used in our study.

Characteristic	Conventional fenestrated cuffed tube (Shiley size 8)	Extended adjustable‐length uni‐cuff tube (Spiraflex size 9)
Inner diameter (ID mm)	7.6	9.0
Outer diameter (OD mm)	12.2	12.2
Tube length (mm)	79	Adjustable, ~155
Flange	Fixed	Adjustable
Cuff	Standard HVLP cuff	Larger, more compliant HVLP cuff
Utility in wide trachea	Often requires high cuff volumes; prone to leak	Allows distal positioning and improved sealing with less reliance on overinflation

Abbreviations: HVLP: high volume low pressure, mm: millimeters.

Although the patient remained clinically stable, early intervention was pursued to prevent potential deterioration, with the primary objective of controlling the source of air leakage. The initial tracheostomy tube was a conventional fenestrated cuffed tube with a smaller inner diameter (7.6 mm) but a comparable outer diameter (12.2 mm) to the subsequently used adjustable‐length, wire‐reinforced tube, which provided a larger inner diameter (9.0 mm). This highlights an important manufacturer‐dependent consideration: a larger tracheostomy tube size does not necessarily correspond to a larger outer diameter, as inner and outer dimensions vary across designs. Thus, in patients with enlarged tracheas, airflow and sealing efficacy are influenced by factors beyond tube caliber.

The principal advantage of the adjustable‐length design lies in its ability to optimize intratracheal positioning, allowing the cuff to be positioned distal to the most dilated airway segment and reducing reliance on excessive cuff inflation. Additionally, these tubes are typically equipped with larger, more compliant, high‐volume cuffs, which better accommodate tracheomegaly and have been shown to reduce refractory air leak when standard‐length tubes require high cuff inflation volumes [13, 17]. Accordingly, the benefit of adjustable tracheostomy systems in this setting appears to be driven primarily by positional flexibility and cuff geometry rather than tube diameter alone [18].

This approach proved successful, as evidenced by the gradual resolution of SE. To our knowledge, this is one of the first reports on massive SE caused by trachea‐tracheostomy tube size mismatch, combined with a fenestration‐associated localized subcutaneous breach, successfully managed through exchange to a non‐fenestrated, adjustable length wire‐reinforced cuffed tracheostomy tube as a primary strategy for addressing the massive post‐tracheostomy SE.

## 4 Conclusion

Massive SE following tracheostomy is an uncommon but potentially serious complication, particularly when associated with pneumomediastinum. This case highlights the importance of identifying possible anatomical and device‐related contributors to persistent air leakage, including tracheal caliber mismatch, fenestrations, and subtle stomal dehiscence. When conservative measures fail, early targeted intervention is warranted. In patients with tracheomegaly, the use of a non‐fenestrated, flexible, wire‐reinforced tracheostomy tube with an adjustable length provided an effective seal, optimized intratracheal positioning, and led to complete resolution without additional invasive procedures. Careful tube selection and fit should be considered central to the management algorithm for massive post‐tracheostomy SE.

## Author Contributions

**Omar Aboul Hosn:** conceptualization, data curation, investigation, methodology, visualization, writing – original draft. **Rita Khattar:** conceptualization, investigation, methodology, writing – original draft. **Gabriel Dunya:** investigation, supervision, writing – review and editing. **Sami Jean Melki:** conceptualization, investigation, supervision, visualization, writing – review and editing. **Jaafar A. Hadi:** writing – review and editing.

## Funding

The authors have nothing to report.

## Ethics Statement

An approval from the Institutional Review Board of the American University of Beirut was not needed for this type of study.

## Consent

Written informed consent was obtained from the patient.

## Conflicts of Interest

The authors declare no conflicts of interest.

## Data Availability

The data that support the findings of this study are available upon request.

## References

[1] I. Baiu and L. Backhus , “What Is a Tracheostomy?,” Journal of the American Medical Association 322, no. 19 (2019): 1932, 10.1001/jama.2019.14994. 31742632

[2] S. Fernandez‐Bussy , B. Mahajan , E. Folch , I. Caviedes , J. Guerrero , and A. Majid , “Tracheostomy Tube Placement: Early and Late Complications,” Journal of Bronchology & Interventional Pulmonology 22, no. 4 (2015): 357–364, 10.1097/LBR.0000000000000177. 26348694

[3] M. C. Stock , C. G. Woodward , B. A. Shapiro , R. D. Cane , V. Lewis , and B. Pecaro , “Perioperative Complications of Elective Tracheostomy in Critically Ill Patients,” Critical Care Medicine 14, no. 10 (1986): 861–863, 10.1097/00003246-198610000-00005. 3757526

[4] K. Kukuruza and A. Aboeed , Subcutaneous Emphysema(Archived). [Updated 2023 Jul 17], StatPearls [Internet] (StatPearls Publishing, 2025), https://www.ncbi.nlm.nih.gov/books/NBK542192/. 31194349

[5] L. F. Berg , M. F. Mafee , M. Campos , and E. L. Applebaum , “Mechanisms of Pneumothorax Following Tracheal Intubation,” Annals of Otology, Rhinology, and Laryngology 97, no. 5 Pt 1 (1988): 500–505, 10.1177/000348948809700512. 3178101

[6] M. J. Mostert and H. Stuart , “Subcutaneous Emphysema Caused by a Fenestrated Tracheostomy Tube,” Anaesthesia 56, no. 2 (2001): 191–192, 10.1046/j.1365-2044.2001.01870-16.x. 11167497

[7] D. M. Kaylie and M. K. Wax , “Massive Subcutaneous Emphysema Following Percutaneous Tracheostomy,” American Journal of Otolaryngology 23, no. 5 (2002): 300–302, 10.1053/ajot.2002.124192. 12239698

[8] S. Saeed , S. Alothman , A. Safavi , B. Donaldson , A. Ramcharan , and H. DePaz , “Tracheostomy Exchange Resulting in Rare Combination of Pneumomediastinum, Pneumothorax, Massive Pneumoperitoneum, and Subcutaneous Emphysema,” Cureus 9, no. 7 (2017): e1489, 10.7759/cureus.1489. 28944128 PMC5602445

[9] J. A. Showmaker and M. P. Page , “Life‐Threatening Tension Subcutaneous Emphysema as a Complication of Open Tracheostomy,” Otolaryngology and Head and Neck Surgery 142, no. 4 (2010): 628–629, 10.1016/j.otohns.2009.09.023. 20304293

[10] Y. N. Kim , Y. S. Chang , K. R. Cho , and B. Y. Kim , “A Case of Massive Subcutaneous Emphysema and Pneumomediastinum due to Dehiscence of Stoma After Emergent Tracheostomy,” Ear, Nose, & Throat Journal 102, no. 5 (2023): 307–311, 10.1177/01455613221129435. 36124380

[11] A. Alshoubi and A. Mathew , “Subcutaneous Emphysema Following Open Tracheostomy During Tracheostomy Mask Ventilation,” American Journal of Case Reports 23 (2022): e937102, 10.12659/AJCR.937102. 36065149 PMC9465497

[12] K. O. Elkholy , H. Akhtar , E. Landa , Y. Malyshev , and S. Sahni , “A Case of Pneumomediastinum and Pneumoperitoneum With Concurrent Massive Subcutaneous Emphysema due to Repositioning of a Tracheostomy Tube,” Cureus 11, no. 1 (2019): e3881, 10.7759/cureus.3881. 30899632 PMC6420328

[13] E. Krustins , “Mounier‐Kuhn Syndrome: A Systematic Analysis of 128 Cases Published Within Last 25 Years,” Clinical Respiratory Journal 10, no. 1 (2016): 3–10, 10.1111/crj.12192. 25130790

[14] N. Fazlıoğlu , H. Sasani , M. Fazlıoğlu , E. P. Çiftçi , and L. C. Mutlu , “Association of Tracheal Diameter With Respiratory Function and Fibrosis Severity in Idiopathic Pulmonary Fibrosis Patients,” BMC Pulmonary Medicine 25, no. 1 (2025): 157, 10.1186/s12890-025-03624-x. 40188355 PMC11972493

[15] T. Berlet and M. Marchon , “Leakage Characteristics of Dual‐Cannula Fenestrated Tracheostomy Tubes During Positive Pressure Ventilation: A Bench Study,” Anesthesiology Research and Practice 2016 (2016): 9272865, 10.1155/2016/9272865. 27073395 PMC4814635

[16] L. Pryor , A. Bassiouni , O. Dale , et al., “A Randomised Crossover Trial Examining the Perceived Clinical Benefits of Fenestrated Tracheostomy Tubes in Head and Neck Patients,” Australian Journal of Otolaryngology 7 (2024): 11, 10.21037/ajo-23-15.

[17] B. Cheon , J. H. Lee , J. H. Kim , and S. M. Hwang , “Airway Management of a Patient With Mounier‐Kuhn Syndrome During General Anesthesia ‐ A Case Report,” Anesthesia and Pain Medicine 19, no. 2 (2024): 156–160, 10.17085/apm.23172. 38725171 PMC11089299

[18] D. R. Hess and N. P. Altobelli , “Tracheostomy Tubes,” Respiratory Care 59, no. 6 (2014): 956–973, 10.4187/respcare.02920. 24891201

